# A probabilistic forecasting framework for neighbourhood-level disaggregation of electric vehicle adoption scenarios

**DOI:** 10.1038/s41467-026-74155-0

**Published:** 2026-06-13

**Authors:** Isaac Flower, Furong Li, Julian Padget

**Affiliations:** 1 Department of Electronic & Electrical Engineering, University of Bath, Bath, UK; 2 Department of Computer Science, University of Bath, Bath, UK

**Keywords:** Energy supply and demand, Electrical and electronic engineering, Energy grids and networks, Applied mathematics

## Abstract

The rapid growth of electric vehicle (EV) adoption presents significant challenges for electricity networks, particularly at the low-voltage level, where clustered neighbourhood demand risks overloading infrastructure. Existing scenario-based planning approaches typically assume uniform EV uptake across neighbourhoods within a region, failing to capture the heterogeneity in historical EV registration data. They provide limited uncertainty quantification, despite the difficulty of predicting future adoption at fine spatial scales. This paper introduces a Gaussian process (GP)-based forecasting framework that combines granular historical EV registration data with top-down regional scenarios to generate probabilistic neighbourhood-level forecasts. The GP captures how local adoption deviates from regional trends, encoded in the GP’s mean function, ensuring consistency with broader scenarios while accounting for local variation and uncertainty. We validate the framework using ten representative local authority districts in England and Wales, covering 1,294 neighbourhoods. The framework demonstrates improved performance compared to baseline methods (scaled scenario, logistic growth, linear extrapolation) in normalised mean absolute error, with statistically significant improvements at horizons of three years and beyond. It also delivers well-calibrated prediction intervals, providing reliable uncertainty estimates. This framework offers a practical tool for network operators, policymakers, and planners to support targeted decision-making and investment.

## Abstract (web summary)

A Gaussian process framework integrates neighbourhood EV registration data with regional scenarios to generate probabilistic local adoption forecasts with uncertainty estimates for electricity network planning.

## Introduction

The rise in electric vehicle (EV) adoption, driven by government mandates and proposed bans on new fossil fuel-powered cars^1,2^, is creating unprecedented challenges for the energy systems around the world^3^. In particular, the need to supply additional electricity for EV charging is placing increasing pressure on electricity networks that were not designed to support the mass electrification of transport. While transmission networks benefit from geographic diversity and demand aggregation, distribution networks-especially at the low-voltage (LV) level-are more vulnerable to capacity constraints due to clusters of EV adoption driving localised load growth^4^. Therefore, network operators, charge point providers, and policymakers must anticipate where, when and how these adoption hot spots will occur to ensure sufficient network capacity and charging infrastructure availability.

Existing approaches to network planning for EV adoption typically use scenarios as top-down narratives to explore plausible long-term system trajectories under uncertainty^5^. These scenarios capture high-level structural, behavioural, and policy uncertainties at national or regional scales, where behaviour aggregates smoothly, and policy signals dominate. However, they fail to reflect the heterogeneity of EV adoption at the neighbourhood level, where EVs connect to and interact with the electricity network. Moreover, manually tailoring scenarios for thousands of small areas is impractical and susceptible to modeller bias with poor empirical grounding.

In the UK, administrative regions such as Local Authority Districts (LADs) are commonly used for scenario-based planning because they align with local government structures, enabling coordination with councils to reflect area-specific policies and plans^6^. At the time of writing, there are 361 LADs in the UK^7^. Recent improvements in the spatiotemporal granularity of reported EV registration data^8^ now enable insights to be drawn from neighbourhood-level areas such as Lower Layer Super Output Areas (LSOAs). LSOAs are small statistical areas comprising between 400 and 1200 households and a population of 1000–3000 people^9^. An LAD will typically contain between 50 and 300 LSOAs^10^. There are over 30,000 LSOAs in England and Wales. However, local adoption patterns are often noisy and highly variable, making it difficult to identify the long-term trends necessary for effective forecasting. To address this, we need a method that links high-level scenarios with granular data-bridging top-down planning with bottom-up empirical evidence, while also providing a principled estimation of the uncertainty surrounding these disaggregated forecasts.

The current EV adoption forecasting approaches proposed in the literature, including: (i) diffusion and growth models^11–17^, (ii) agent-based models^18–20^, (iii) system dynamics models^21–27^, and (iv) statistical or machine learning models^28–31^ are not well-positioned to fill this gap. These models generally suffer from poor accuracy and limited validation^32^, low spatial granularity (rarely applied below national or regional levels), and inadequate uncertainty quantification. In this paper, we argue that Gaussian processes (GPs) offer a flexible probabilistic framework to address these challenges. GPs are a non-parametric statistical method that defines distributions over functions rather than assuming a fixed parametric form. They are well-suited to the task of forecasting EV adoption, where we expect non-linear trends, have limited historical data, and a need to quantify predictive uncertainty. GP models are not typically applied to long-term forecasting because they tend to revert to the prior mean and exhibit growing uncertainty beyond the range of observed data. However, in this paper, we argue that by incorporating prior knowledge through a non-zero mean function or a non-stationary kernel, GPs can provide informative long-term forecasts. This approach has been applied in other contexts, such as Richardson et al.^33^, who use a separate battery degradation model as the mean function and a GP to capture residual variation.

In this work, we present a forecasting framework that uses GPs to predict how neighbourhood-level EV adoption will deviate from regional trends, based on historical observations. This enables scenario-consistent forecasts that still account for local variability and uncertainty. The framework generates coherent, probabilistic neighbourhood-level forecasts conditioned on regional scenarios. It allows decision-makers to ask: If a particular scenario unfolds, what is the likely distribution of outcomes at the neighbourhood level? Or conversely: How might different neighbourhoods collectively contribute to a regional adoption trajectory? The framework combines several key methodological innovations. It leverages neighbourhood-level EV registration data to capture local heterogeneity, incorporates regional EV adoption scenarios as custom GP mean functions to ensure consistency with top-down planning, and spatially disaggregates regional scenarios into probabilistic neighbourhood-level forecasts. A probit transformation is also applied within the GP framework to produce bounded forecasts that respect the [0, 1] domain of EV market share. We apply the proposed framework to EV adoption in England and Wales, where LAD-level EV adoption scenarios are spatially disaggregated into probabilistic neighbourhood-level forecasts using historical LSOA vehicle registration data. A two-step k-means clustering process is used to systematically characterise LADs based on historical EV adoption patterns across their constituent LSOAs. The framework is evaluated through rigorous multi-horizon validation across 1294 LSOAs using both deterministic and probabilistic forecasting metrics, providing a clear benchmark for future research on spatially granular EV adoption forecasting.

## Results

### The forecasting framework

**Fig. 1:**
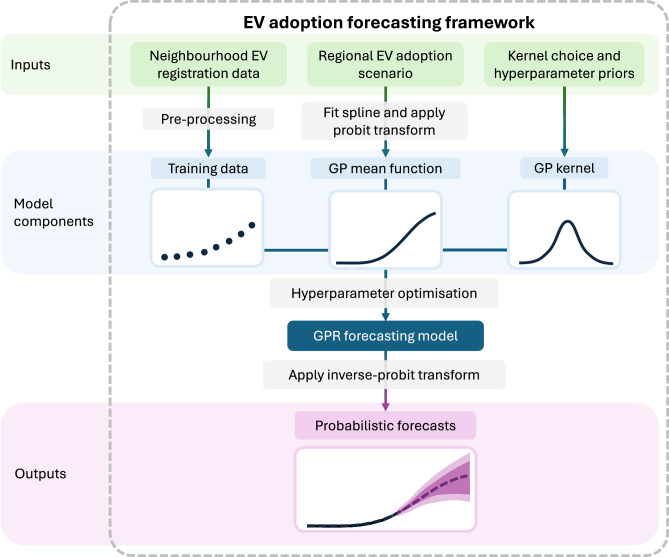
EV adoption forecasting framework. The framework disaggregates regional EV adoption scenarios into spatially granular, probabilistic forecasts of neighbourhood-level EV market share (EVMS). Inputs include neighbourhood-level EV registration data, regional adoption scenarios, and Gaussian process (GP) kernel specifications. These are combined through a GP model with a custom mean function, producing probabilistic forecasts that can be interpreted through prediction intervals. The framework enables estimation uncertainty in EV adoption and supports network planning decisions by providing localised forecasts consistent with regional scenarios. Illustrative plots are shown for conceptual purposes only and do not represent actual model outputs.

The central idea of the framework is to disaggregate regional-level EV adoption scenarios into local neighbourhoods. The ability to translate scenarios into spatially granular, probabilistic forecasts is valuable for applications such as predicting the impact of EV charging on electricity distribution networks, providing richer evidence and confidence for distribution system operators (DSOs) to invest ahead of time. Figure 1 provides an overview of the EV adoption forecasting framework. The inputs, model components and outputs are described in the following sections.

The framework has three key inputs: (i) Neighbourhood-level EV registration data, (ii) a regional EV adoption scenario, and (iii) the choice of GP kernel and its hyperparameter priors.

This paper represents EV adoption using the proportion of registered vehicles that are EVs, referred to as EV market share (EVMS), which is defined on the interval [0, 1]. This measure allows for direct comparison across areas of varying size and vehicle stock, and aligns with common definitions in the literature. Details of the datasets used for applying the framework to LSOAs and LADs in England and Wales, the data preprocessing steps undertaken, and the specific choice of hyperparameter priors are described in the Methods section.

GPs sit at the core of the framework, allowing for principled integration of spatially granular EV adoption data (as training data) and regional scenarios (as the mean function). Assumptions about future EV adoption dynamics are embedded in the GP kernel hyperparameters. A formal definition of a GP is given in the Methods section. There are three main components to a GP model: (i) The kernel, (ii) the mean function, and (iii) the training data.

Selecting an appropriate kernel for a GP model is inherently subjective and can significantly impact the model’s performance and interpretability. In this work, we use a Radial Basis Function (RBF) kernel. Further details of the RBF kernel and justification of its use can be found in the Methods section.

The mean function provides a foundation from which the kernel captures the latent function’s characteristics. While often assumed to be zero, our framework incorporates regional EV adoption scenarios as a custom mean function. In this study, we use historical regional EVMS trajectories as idealised best-case scenarios. This design isolates the disaggregation model’s performance from scenario-level uncertainty, so forecast errors reflect the model’s ability to capture spatial heterogeneity. The process of creating a custom mean function for each region is further described in the Methods section.

The training data provides the observed evidence on which the GP is conditioned, enabling it to learn how neighbourhood-level EV adoption has historically evolved and the level of diversity across the region. This component captures local variation and informs the posterior distribution around the mean function. To constrain the GP’s outputs between 0 and 1, a probit transformation is applied to the training data and the custom mean function before conditioning. The inverse probit function can revert the GP output to the original EVMS domain. Further details on the probit transformation, model training, and hyperparameter optimisation can be found in the Methods section.

A probabilistic forecast is generated by drawing 1000 posterior GP samples, representing possible future trajectories. This means that for each forecast year, the predicted EVMS is expressed as a set of 1000 samples. These samples are then used to construct prediction intervals (PIs), which indicate the probability that the true value lies within a given range. Further details and justification for this approach are provided in the Methods section.

### Characterisation of local EV adoption in England and Wales

We adopt a two-step k-means clustering approach to characterise LADs based on their historical EV adoption. This involves: (i) Clustering LSOAs based on historical EV adoption. (ii) Clustering LADs based on their composition of LSOAs from different clusters.

**Fig. 2:**
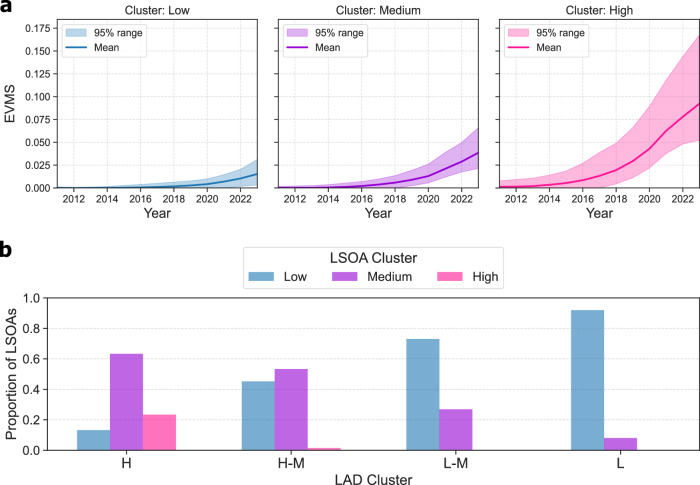
Clustering of local EV adoption trends in England and Wales. **a** Lower-layer Super Output Areas (LSOAs) are grouped into three clusters based on historical EV adoption trajectories, representing low-, medium-, and high-adoption profiles. The lines show the mean EV market share (EVMS) for each cluster, and the shaded areas indicate the 95% range across LSOAs. **b** Local Authority Districts (LADs) are clustered according to the composition of their constituent LSOAs across the three adoption profiles, forming four distinct LAD clusters (H, H-M, L-M, and L). The bars show the average LSOA composition for each LAD cluster.

Using k-means clustering, LSOAs in England and Wales were grouped into three clusters, based on their historical EV registration data. These broadly represent low, medium, and high EV adoption profiles. Figure 2a shows the mean and 95% range for each cluster. Overall, 70.2% of LSOAs were grouped into the low-adoption cluster, 27.1% into the medium-adoption cluster, and 2.7% into the high-adoption cluster.

The 331 LADs in England and Wales were clustered based on the proportion of their constituent LSOAs belonging to each EV adoption profile cluster. Using k-means clustering, four distinct LAD clusters were identified, labelled H, H-M, L-M, and L. The average LSOA composition of each cluster is illustrated in Fig. 2b. Cluster H LADs (8.5% of total) are characterised by a predominance of medium-adoption LSOAs, along with a notable share of high-adoption and some low-adoption LSOAs. Cluster H-M (18.4% of total) consists of LADs with a relatively balanced mix of low- and medium-adoption LSOAs, with a small amount of high-adoption LSOAs. Cluster L-M LADs (25.4% of total) consist mostly of low-adoption LSOAs, with a smaller proportion of medium-adoption LSOAs. Cluster L (47.7% of total) includes LADs made up almost entirely of low-adoption LSOAs, with only a few medium-adoption LSOAs.

### Selecting representative LADs

Due to the high computational cost of applying the forecasting model to all LSOAs in England and Wales, we instead evaluate the framework on a representative subset. Two LADs were selected from each cluster to evaluate model performance across a representative range of EV adoption patterns. For each cluster, the LADs at the 25th and 75th percentiles of the 2023 EVMS were chosen, ensuring a broad spread of EV adoption levels. Wandsworth and Hackney were selected from cluster H, Hillingdon and Harrogate from cluster H-M, South Gloucestershire and Charnwood from cluster L-M, and Crawley and Dudley from cluster L.

Additionally, LADs with the highest and lowest EVMS in 2023 were included as outliers. As the LAD with the highest EVMS (City of London) contained only five LSOAs, the next highest LAD (Westminster) was selected to allow for a more meaningful evaluation. Blaenau Gwent was the LAD with the lowest EVMS in 2023.

**Fig. 3:**
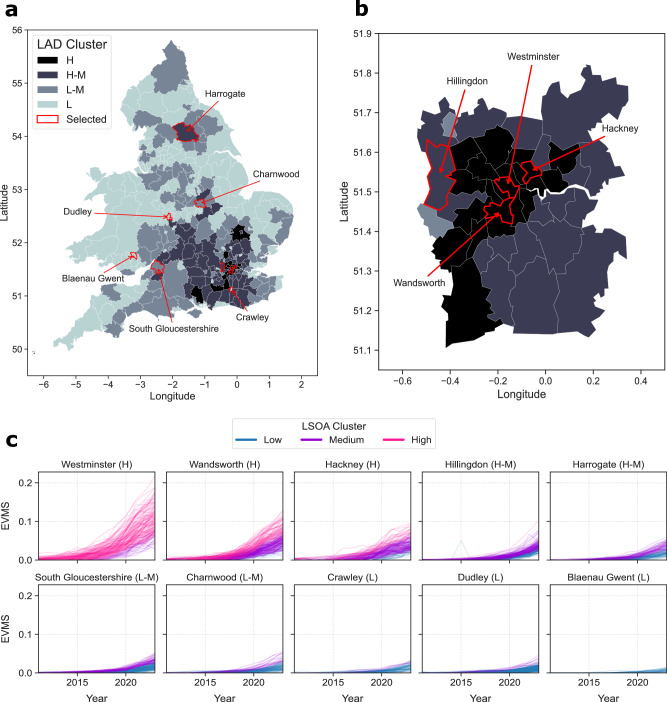
Overview of selected LADs and their cluster membership. **a** Local Authority Districts (LADs) in England and Wales coloured by EV adoption cluster, with the ten selected LADs highlighted in red. **b** Close-up view of selected London LADs. **c** Historical EV market share (EVMS) trajectories of all Lower-layer Super Output Areas (LSOAs) within each selected LAD, coloured by their LSOA cluster membership. The figures use boundary data from the Office for National Statistics licensed under the Open Government Licence v.3.0. Contains OS data © Crown copyright and database right [2023].

Figure 3a shows the LADs in England and Wales, coloured by cluster, with the ten selected LADs highlighted in red. A zoomed-in view of the selected LADs in the London area is provided in Fig. 3b. The distribution of clusters in Fig. 3a highlights regional disparities in EV adoption. LADs in the south of England generally contain a higher proportion of high- and medium-adoption LSOAs compared to those in Wales and the North. Most LADs in cluster H are located in London, while Wales is almost entirely composed of LADs assigned to cluster L. These spatial patterns underscore the uneven distribution of EV adoption across the country. Figure 3c plots the LSOA-level EV adoption profiles within each of the selected LADs, which are coloured according to their respective LSOA clusters.

Table 1 displays key summary statistics for the representative LADs in 2023, illustrating differences in LAD size (number of LSOAs) and levels of vehicle and EV ownership.

**Table 1:** Summary of 2023 EV adoption in the study area

LAD	No. LSOAs	Vehicles	EVs	EVMS (%)
Westminster	128	283 [224–366]	25 [19–42]	9.7 [7.4–12.4]
Wandsworth	179	400 [330–467]	22 [14–32]	5.5 [4.2–7.3]
Hackney	144	260 [221–293]	10 [7–14]	3.8 [3.0–5.3]
Hillingdon	161	699 [644–788]	24 [18–31]	3.4 [2.7–4.0]
Harrogate	104	778 [678–954]	20 [12–34]	2.5 [1.7–3.7]
South Gloucestershire	165	853 [750–1002]	17 [11–30]	2.1 [1.3–2.9]
Charnwood	99	841 [684–1068]	14 [8–27]	1.8 [1.0–2.7]
Crawley	66	750 [657–849]	12 [7–18]	1.7 [1.0–2.2]
Dudley	201	753 [664–834]	9 [6–14]	1.2 [0.8–1.7]
Blaenau Gwent	47	667 [616–778]	5 [3–7]	0.8 [0.4–1.0]

Number of Lower Layer Super Output Areas (LSOAs) within each selected Local Authority District (LAD), together with the median and interquartile range (IQR) of LSOA-level total vehicles, EVs, and EV market share (EVMS). IQR values are shown in square brackets.

### Data availability across selected LADs

**Fig. 4:**
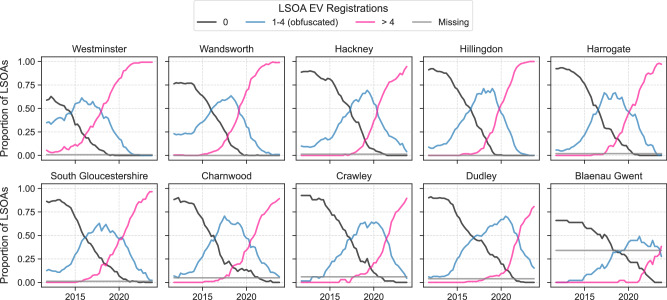
LSOA-level data availability for selected LADs. Proportion of Lower-layer Super Output Areas (LSOAs) within each selected Local Authority District (LAD) by EV registration data category from 2011 to 2023: no data reported, zero EVs, obfuscated counts (1–4 EVs), and five or more EVs. The figure shows how data availability has improved over time as EV adoption increases, with most LSOAs reaching reliable reporting by 2023. Blaenau Gwent is an exception, with a high share of LSOAs lacking registration data entirely.

Figure 4 shows the proportion of LSOAs within each selected LAD by the level of EV registration data availability, before any data processing, imputation or interpolation. In 2011, nearly all LSOAs had no registered EVs. As adoption increased over time, the number of LSOAs with zero EVs declined, but many transitioned into the obfuscated category between 1 and 4 EVs, for which the DfT withholds exact counts. The peak level of obfuscated data occurs from around 2017 for high-adoption LADs to 2020 for low-adoption LADs.

As adoption continues, the proportion of LSOAs with 5 or more registered EVs increases steadily, reaching nearly 100% by the end of 2023 in most areas. This trend indicates that reliable registration data is now available for the vast majority of LSOAs. One exception is Blaenau Gwent, where a large proportion of LSOAs have no EV registration data reported at all-neither zero nor obfuscated-highlighting a lag in both EV uptake and data availability.

### Forecasting performance

We evaluate the model’s predictive performance by simulating how it would have performed on historical data. To do this, we train five versions of the model, each using data available up to a different year: *t*_*n*_ = 2018, 2019, 2020, 2021 and 2022. For each version, we assess how well it predicts outcomes in the subsequent years (*t*_*f*_), up to 2023. The forecast horizon, *h*_*f*_, is defined as the number of years into the future for which a prediction is made, relative to the final year of training data.

We limit our evaluation to forecasts with horizons of five years or less. Beyond this range, the scarcity of historical data and the prevalence of neighbourhoods with zero EVs introduce substantial uncertainty, making such evaluations an unreliable reflection of the model’s long-term predictive performance. We compare our model’s deterministic performance against three baseline approaches: (i) Scaled scenario (ii) Logistic growth (iii) Linear extrapolation.

The details of these baseline models are described further in the Methods section.

**Fig. 5:**
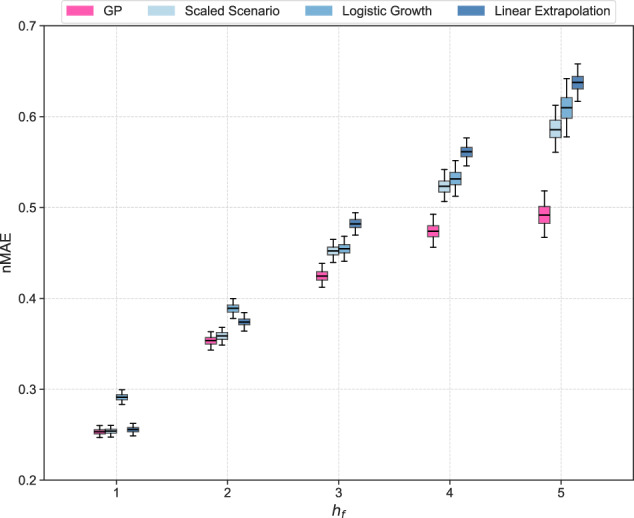
Forecasting performance across baseline models. Box plot comparing normalised mean absolute error (nMAE) of the Gaussian process (GP) model against three baseline approaches (Scaled Scenario, Logistic Growth, and Linear Extrapolation) across forecast horizons, *h*_*f*_, from one to five years. The nMAE is calculated over all Lower-layer Super Output Areas (LSOAs) in each of the ten representative Local Authority Districts (LADs). The centre line represents the median, the box represents the interquartile range, and the whiskers indicate the 2.5th and 97.5th percentiles. These percentile bounds are estimated via bootstrap resampling, where the original set of errors is resampled with replacement *n* = 1000 times and the nMAE is recomputed for each resample to form an empirical distribution. The GP model consistently achieves lower nMAE values than baseline approaches, with the largest performance gains observed at longer forecast horizons.

Figure 5 compares the normalised mean absolute error (nMAE) of the GP model against these three baseline approaches, computed across LSOAs within all selected LADs. The GP model consistently achieves lower or comparable nMAE values, with statistically significant improvements (*p* < 0.05) over all baselines at forecast horizons of three years and beyond. This indicates its ability to more accurately capture long-term neighbourhood-level EV adoption patterns compared to simpler extrapolation or scaling-based methods.

The performance gap becomes more pronounced at longer horizons, reflecting the GP model’s flexibility and its ability to borrow strength from the underlying LAD-level scenario. As the forecast horizon extends, the GP’s predictions gradually converge toward the LAD mean function, which serves as a reasonable approximation of average LSOA-level behaviour. In contrast, the simpler baselines do not benefit from the regularising effect of the LAD-level mean function used by the GP model, leading to larger forecast errors over time. While the Scaled Scenario incorporates the LAD-level scenario as prior information, it assumes that the historical proportional difference between LSOA- and LAD-level EV adoption remains constant over the entire forecast horizon.

Here, we provide a more detailed evaluation of the GP model’s forecasting accuracy across multiple forecast horizons for each LAD. To assess how much the proposed framework improves forecast accuracy, we use the idealised LAD-level scenario as a performance benchmark. However, since this scenario is not learned from data and remains fixed over time, it does not produce forecasts in the conventional sense-there is no notion of a forecast horizon, and it is not updated as new data becomes available. For this reason, the LAD-level scenario is only used as a benchmark for a given target year, rather than as a competing forecasting model across different forecast starting points with limited access to training data.

**Fig. 6:**
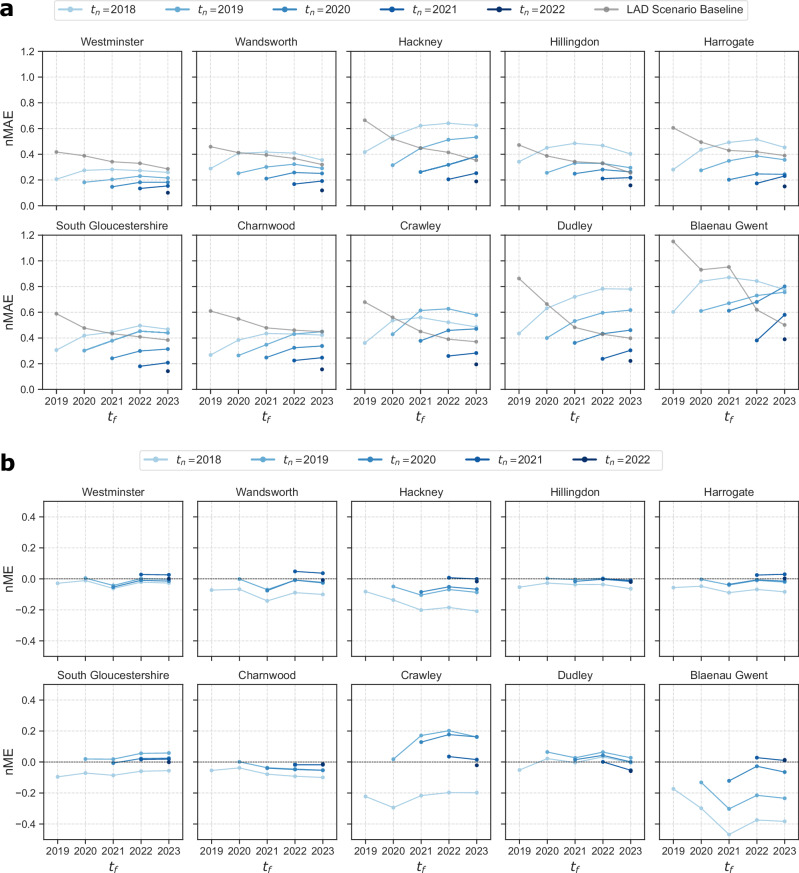
Forecast accuracy and bias across selected LADs. **a** Normalised mean absolute error (nMAE) and **b** Normalised mean error (nME) of Lower Layer Super Output Area (LSOA)-level forecasts for each Local Authority District (LAD), evaluated across multiple forecast starting points (*t*_*n*_) and target years (*t*_*f*_). Shades of blue represent different forecast starting points, where forecasts made in *t*_*n*_ = 2018 are evaluated for *t*_*f*_ ∈ {2019, 2020, 2021, 2022, 2023}. Grey lines show the nMAE of the idealised LAD-level scenario used as a benchmark; values below this baseline indicate improved deterministic accuracy from spatial disaggregation. Deviations of nME from zero indicate systematic model bias, where positive values reflect over-prediction and negative values reflect under-prediction of EV adoption.

Figure 6a presents the nMAE for each LAD across multiple forecast horizons and starting points. A grey baseline representing the LAD scenario is included for reference, highlighting the extent to which the GP model adds predictive value through spatial disaggregation. When the model’s trace lies below the baseline, it provides a more accurate deterministic forecast than the top-down LAD scenario. Conversely, if the trace rises above the baseline, the model offers no improvement in predictive accuracy over the baseline.

Overall, the results show that forecast accuracy improves over time for a given forecast horizon-that is, forecasts generated from later years tend to be more accurate than those from earlier years. This reflects the value of incorporating additional historical data, which allows the autoregressive GP model to better learn underlying adoption patterns through improved kernel hyperparameter estimation. As a result, the model can more effectively distinguish persistent local deviations from short-term noise, leading to better forecasting accuracy. The LAD scenario baseline accuracy also improves over time. As EV adoption increases between 2019 and 2023, the nMAE of the LAD scenario decreases across all LADs, reflecting a gradual convergence of neighbourhood-level EV adoption towards the regional trend as uptake rises.

Models trained on data up to 2018 and 2019 initially outperform the LAD baseline but begin to underperform at longer horizons. This indicates a tendency for models to overfit to early historical trends, which results in forecasts becoming misinformed about the future trajectory of EV adoption. As more training data becomes available, this overfitting effect diminishes. Models trained from 2020 onwards consistently outperform the LAD baseline across all forecast horizons, with a few exceptions found in the low-EVMS LADs from cluster L (Crawley, Dudley and Blaenau Gwent). Notably, forecasts for LSOAs within Westminster, the LAD with the highest EV market share-exceed the LAD scenario’s performance for all starting points and horizons.

While the long-term accuracy of the GP model is difficult to evaluate definitively, the results suggest that its deterministic forecasts (based on the predictive mean) offer limited advantages beyond horizons of several years ahead, particularly in areas with low historical EV adoption. Nevertheless, in data-rich settings like Westminster, the model demonstrates strong potential, indicating that performance is likely to improve further as more complete data becomes available.

Figure 6b visualises systematic errors (bias) in the GP model’s forecasts for each LAD by tracking the normalised Mean Error (nME) across different horizons and starting points. The results indicate minimal model bias for most LADs, with nME values remaining close to zero. The lack of systematic bias across most LADs is likely a consequence of anchoring the GP mean function to the observed LAD-level EVMS. This encourages local forecasts to remain centred on the regional trend unless the LSOA-level data provides strong evidence for persistent deviation. If the LAD-level EVMS scenarios were inaccurate, this anchoring mechanism would directly propagate systematic bias into the local forecasts.

However, there are several notable exceptions. In Blaenau Gwent, forecasts tend to underestimate EVMS, as indicated by predominantly negative nME values. This behaviour is likely driven by the presence of many LSOAs with zero registered EVs prior to 2019, which pulls forecasts below the LAD-level scenario as the model extrapolates this zero-adoption pattern forward. Over time, as EV adoption becomes non-zero in more LSOAs, the nME gradually converges towards zero, in line with other LADs experiencing earlier adoption.

A related but contrasting pattern is observed in Crawley. The forecasts trained on data up to 2018 (*t*_*n*_ = 2018) initially face a similar issue of widespread zero-EV LSOAs. However, when data from 2019 is incorporated (*t*_*n*_ = 2019), the nME suddenly becomes positive. This reflects a sharp increase in EV adoption in Crawley during 2019, which the model extrapolates forward, leading to systematic overestimation when this growth does not persist at the same rate.

Wandsworth and Hackney exhibit similar, though less pronounced, behaviour, with slightly negative nME values for early forecasts that converge back towards zero as EV adoption becomes more widespread across LSOAs. The behaviour highlights the challenges of forecasting early-stage EV adoption where historical data is limited.

To assess the model’s ability to quantify uncertainty, we use the prediction interval coverage probability (PICP). A prediction interval (PI) specifies the range within which the true outcome is expected to fall with a given probability, providing a measure of uncertainty around the forecast. PIs can be used to quantify risks and inform more robust planning decisions by providing bounds on future outcomes rather than relying solely on point forecasts. We visualise the model’s calibration by plotting the observed coverage against the nominal PI level. A well-calibrated model will produce a near-diagonal line, indicating that the observed coverage aligns closely with the intended probability levels.

**Fig. 7:**
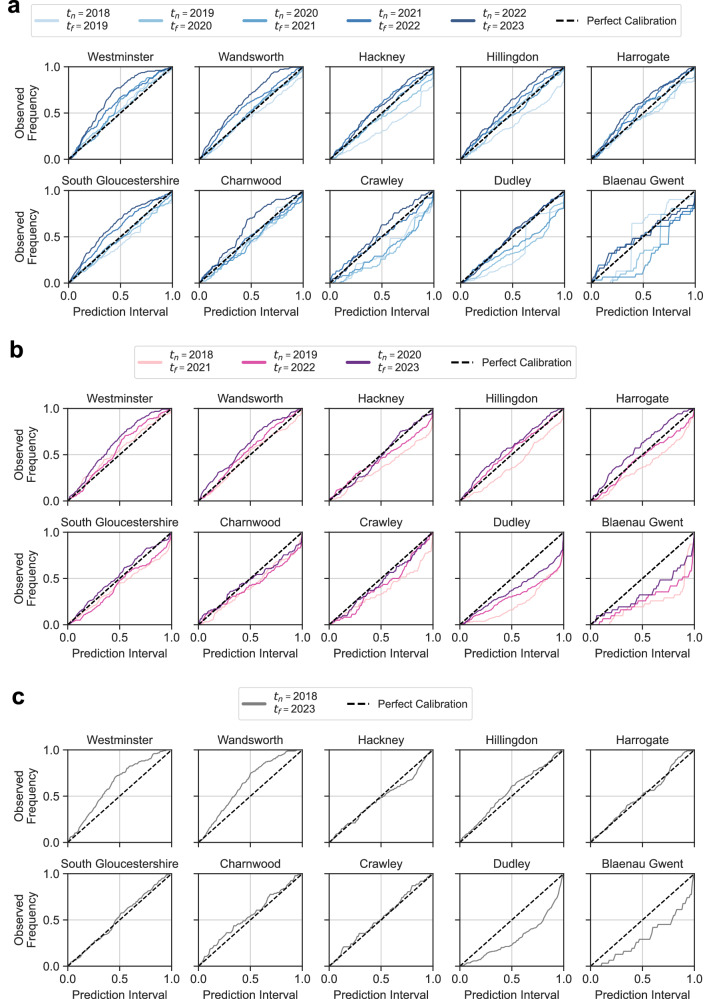
Calibration of forecast uncertainty using PICP. Prediction interval coverage probability (PICP) plots for ten representative Local Authority Districts (LADs) over forecast horizons of **a** *h*_*f*_ = 1 year, **b** *h*_*f*_ = 3 years, and **c** *h*_*f*_ = 5 years. Each trace represents a forecast made at a starting year (*t*_*n*_) and evaluated at a future year (*t*_*f*_). The dashed diagonal denotes perfect calibration, where observed coverage matches the nominal prediction interval (PI) level. Traces above the diagonal indicate conservative forecasts (intervals too wide), while traces below indicate overconfident forecasts (intervals too narrow). Overall, the GP model achieves good calibration across LADs and horizons, demonstrating reliable uncertainty quantification.

Figure 7 shows the model’s PICP for the ten representative LADs across several forecast horizons (*h*_*f*_ = 1, 3, 5), with each trace representing a different forecast start year. Overall, the model’s uncertainty estimates, expressed as PIs, are well calibrated, meaning the intervals reflect the actual likelihood of the true EVMS falling within them. This is evidenced by the close alignment of most traces with the diagonal line, which represents perfect calibration. Importantly, calibration remains relatively stable across all forecast horizons, indicating the robustness of the model’s uncertainty estimates over time.

This good calibration suggests that the model is able to capture the variability of future LSOA-level EV adoption using patterns observed in the historical data. In particular, the GP accounts for two main sources of uncertainty: (i) cross-sectional variability in adoption across LSOAs at a given point in time, reflected in the rate at which LSOA-level trajectories diverge from the LAD-level trend, and (ii) the temporal uncertainty within individual LSOAs, which determines how strongly past observations constrain future adoption trajectories. These uncertainties are encoded in the kernel hyperparameters, which are estimated to best explain the observed spatial and temporal variability in the training data.

The best-calibrated intervals are observed in LADs from clusters H-M and L-M with moderate EV uptake. However, the model’s PIs tend to be overly conservative (i.e. too wide) in high-adoption LADs and overconfident (too narrow) in low-adoption LADs. In areas with very high EV uptake, such as Westminster, the availability of richer training data should improve model performance. This is evidenced by the low nMAE in Fig. 6a. However, the PIs may be too wide due to the close alignment between LSOA- and LAD-level EVMS, resulting in a lower observed coefficient of variation than the model anticipates. This effect may also stem from conservative choices in hyperparameter priors. Conversely, in low-adoption LADs with sparse or flat historical data, such as Blaenau Gwent, the model likely does not observe enough variability in the training data to make predictions that accurately reflect future variability in local adoption trajectories. Taken together, the accuracy, bias, and calibration results demonstrate that forecasting performance is strongly impacted by both historical data availability and the stage of local EV adoption.

## Discussion

This study aimed to develop a probabilistic forecasting framework for neighbourhood-level EV adoption that addresses the limitations of existing scenario-based planning approaches. EV adoption has historically and will likely continue to exhibit substantial variation at the neighbourhood level that cannot be adequately captured by top-down scenarios alone. Deterministic forecasts are inherently limited in their ability to reflect the uncertainty in future EV adoption, especially at the neighbourhood scale. This paper provides a GP-based probabilistic forecasting framework that captures neighbourhood-level heterogeneity in EV adoption by modelling how granular EV registration data deviates from regional scenarios. Our framework significantly outperforms several deterministic baseline approaches (scaled scenario, logistic growth, linear extrapolation) in terms of nMAE at forecast horizons of three years and beyond, with modest gains observed at shorter horizons.

In contrast to traditional deterministic forecasts, the framework produces full probabilistic forecasts with PIs that more accurately represent the range of plausible future scenarios. The framework’s uncertainty estimates are well-calibrated for a range of LADs with differing levels of EV adoption. The framework’s ability to attribute probabilities to potential outcomes makes it well-suited for informing long-term infrastructure planning and policy decisions, offering significant value for a number of stakeholders. DSOs could use these local forecasts to better plan network investments while managing risk more effectively. Charge point operators and local authorities could use these forecasts to target charging infrastructure investments for underserved areas. At the national level, organisations such as the Office of Gas and Electricity Markets (Ofgem, the UK energy regulator) and the National Energy System Operator (NESO, responsible for electricity system operation and planning) could use these forecasts to better understand how regional planning strategies might translate to the neighbourhood level, helping to ensure a more equitable and efficient EV transition. Furthermore, the approach is generalisable to other countries or regions where both top-down scenarios and granular registration data are available.

Data quality and completeness remain a challenge, particularly at the neighbourhood level. A significant portion of the historical EV registration data used to train the models was either missing or obfuscated due to privacy-preserving measures. To address this, imputation and interpolation techniques were applied to recover trends from otherwise incomplete data. Despite these challenges, the framework consistently outperforms the LAD-level scenario in terms of nMAE across short- to medium-term horizons. As the models are trained on more data, the framework maintains this performance advantage over longer forecast horizons, reflecting the value of richer training data in capturing neighbourhood-level dynamics. The reduced benefit at longer horizons when trained on limited data underscores the difficulty of projecting fine-grained spatial heterogeneity based solely on early adoption patterns. As data coverage improves and fewer values fall below obfuscation thresholds, the reliability and precision of disaggregated forecasting methods such as this are expected to increase.

While the forecasted PIs are generally well-calibrated, some variation in calibration is observed at the extremes: PIs tend to be slightly conservative in very high-adoption LADs and overconfident in very low-adoption LADs. The calibration of forecasts for areas with extreme EV adoption could potentially be improved by incorporating PICP directly into the training objective or using Bayesian inference for hyperparameter estimation. While this would increase computational cost, it may substantially improve uncertainty quantification. Future work could explore heteroscedastic GP models that allow observation noise to vary across LADs, providing more flexible and locally tailored uncertainty estimates. There is also scope to enhance forecasting accuracy by incorporating additional features such as socioeconomic variables, housing stock characteristics, or infrastructure availability. Moreover, this could potentially provide deeper insights into the key drivers of EV adoption. Future work could also analyse the impact of inaccurate regional-level scenarios on neighbourhood-level forecasting accuracy. Additional validation of this forecasting framework could be performed using data from high-EV adoption countries such as Norway. Finally, the framework could be used to forecast the adoption of other domestic low carbon technologies, such as heat pumps or rooftop photovoltaic panels, if similar hierarchical adoption data is available.

Overall, the proposed framework offers a practical, data-driven approach to support more targeted, adaptive, and equitable decision-making and investment across the electricity and transport sectors.

## Methods

### Data pre-processing

Table 2 summarises the four datasets used to forecast EV adoption in England and Wales. Datasets VEH0105^34^ and VEH0125^35^ contain the number of licensed vehicles at the end of each quarter for all vehicle types in Local Authority Districts (LADs) and Lower Layer Super Output Areas (LSOAs), respectively. Datasets, VEH0142^7^ and VEH0145^8^, contain similar data but for Battery Electric Vehicles (BEVs) and Plug-in Hybrid Electric Vehicles (PHEVs). While the framework can, in principle, forecast BEV and PHEV adoption separately, in this case study, we combine their registrations (collectively referred to as EVs) to maximise data availability and streamline the presentation of results.

**Table 2:** Datasets used in the analysis

Name	Geography	Vehicle type	Date range
VEH0105^34^	LAD	All vehicles	2009 Q4–2023 Q4
VEH0142^7^	LAD	BEVs and PHEVs	2009 Q4–2023 Q4
VEH0125^35^	LSOA	All vehicles	2011 Q1–2023 Q4
VEH0145^8^	LSOA	BEVs and PHEVs	2011 Q1–2023 Q4
LAD Boundaries^43^	LAD	—	2022
LSOA to LAD Lookup^10^	—	—	—

Vehicle registration datasets for all vehicles, Battery Electric Vehicles (BEVs) and Plug-in Hybrid Electric Vehicles (PHEVs) at Local Authority District (LAD) and Lower Layer Super Output Area (LSOA) levels, together with spatial datasets for LAD boundaries and an LSOA-LAD lookup table.

To avoid discontinuities or anomalous hot spots caused by changes in company car registrations, all datasets are filtered to include only privately owned cars. To protect individual privacy, the Department for Transport (DfT) obfuscates data for LSOAs with between 1 and 4 registered vehicles. While this rarely affects total vehicle registrations, EV registrations at the LSOA level are often significantly impacted, particularly in earlier years when adoption was low. As a result, a substantial portion of the historical EV data is obfuscated rather than truly missing.

We attempt to reconstruct obfuscated data where possible using imputation and interpolation. LSOAs with entirely missing EV registration data (i.e. no data for any time step) are excluded from the analysis.

For partially obfuscated data, the following reconstruction steps are applied. Firstly, if both total and company-owned EV registrations are available for a given quarter and LSOA, the number of privately owned EVs is estimated as the difference between the two. Secondly, if only the total number of EVs is available, the number of privately owned EVs is approximated as half the total. Third, if an LSOA contains some missing values, the first missing data point is set to one to facilitate subsequent interpolation. Finally, any remaining missing data is linearly interpolated using available values from adjacent quarters. This uses the interp() method from the numpy library in Python^36^.

These steps recover more data than linear interpolation alone, especially during early periods with sparse reporting. To reduce noise, the data is annualised by taking the value from the fourth quarter of each year to represent the total vehicle registrations at the end of that year. If an LSOA’s EV market share (EVMS) profile contains many leading zeros (years with no registered EVs), it is truncated to retain only one zero-valued time step at the start. This ensures the profile reflects the onset of adoption without overemphasising the pre-adoption period.

### Clustering EV adoption profiles

For LSOA clustering, each EV adoption profile is encoded as a multi-dimensional vector, where each dimension corresponds to the EVMS in a given year. K-means clustering is applied using Euclidean distance to group similar adoption trajectories. Based on silhouette score analysis, the optimal number of clusters was found to be three, broadly aligning with low, medium, and high EV adoption profiles.

For LADs, clustering is performed based on the composition of their constituent LSOAs across these three EV adoption profile clusters. Since the silhouette scores did not indicate a clear optimum, the number of LAD clusters was manually set to four, prioritising interpretability and ensuring sufficient diversity across the resulting groupings.

### The Gaussian process model

The goal of regression is to learn the underlying latent function that best describes the data-generating process of interest. Numerous methods have been developed to solve regression problems, which can be broadly divided into parametric and non-parametric methods. Parametric methods restrict the class of functions that are considered by assuming a particular functional form. On the other hand, non-parametric methods, such as GPs, are agnostic to the underlying form of the latent function, meaning that they can often accommodate more complex relationships in the data.

While many regression methods aim to learn a single best-fit function, GPs define a probability distribution over possible latent functions that could explain the observed data. This makes them particularly well-suited for sparse or small datasets, where uncertainty quantification is critical. Expert and prior knowledge can be easily incorporated into GPs, making them highly adaptable to various forecasting scenarios. Here, we use GPs to model the evolution of EVMS over time at the neighbourhood level, where each neighbourhood provides a univariate time series of EVMS observations.

Formally, a GP can be defined as a distribution over functions such that, for any finite set of inputs ${{\bf{x}}}={x}_{1},\ldots,{x}_{{N}_{D}}$, the corresponding function values $f({{\bf{x}}})=\left[f({x}_{1}),\ldots,f({x}_{{N}_{D}})\right]$ have a joint Gaussian distribution^37^. A GP is fully specified by its mean function, *m*(**x**), and its covariance function (or kernel), $k({{\bf{x}}},{{{\bf{x}}}}^{{\prime} })$^38^. We define these as

1 $$m({{\bf{x}}})={\mathbb{E}}\left[f({{\bf{x}}})\right]$$

2 $$k({{\bf{x}}},{{{\bf{x}}}}^{{\prime} })={\mathbb{E}}[\left(f({{\bf{x}}})-m({{\bf{x}}})\right)\left(f({{{\bf{x}}}}^{{\prime} })-m({{{\bf{x}}}}^{{\prime} })\right)],$$

and write the GP as

3 $$f({{\bf{x}}}) \sim {{\mathcal{GP}}}(m({{\bf{x}}}),\,k({{\bf{x}}},{{{\bf{x}}}}^{{\prime} })).$$

The mean function is typically assumed to be zero or another simple function. In cases where there is evidence to suggest that the function should take a particular shape (e.g. informed by some physical model or government mandates), GPs can be used to model the residuals. The kernel determines how the model generalises or extrapolates to new data by encoding assumptions about the function’s properties, such as smoothness and periodicity.

To make predictions at new input locations, x*, the joint Gaussian distribution is conditioned on the observed data ${{\bf{y}}}={y}_{1},\ldots,{y}_{{N}_{D}}$. The conditional distribution of *f*(x*) given the training data is:

4 $$f({{{\bf{x}}}}{*})| {{\bf{x}}},{{\bf{y}}},{{{\bf{x}}}}{*} \sim {{\mathcal{N}}}\left(f({{{\bf{x}}}}{*})| {{{\bf{m}}}}{*},{\Sigma }{*}\right),$$

where:

5 $${{{\bf{m}}}}{*}=m({{{\bf{x}}}}{*})+k({{{\bf{x}}}}{*},{{\bf{x}}})k{({{\bf{x}}},{{\bf{x}}})}^{-1}({{\bf{y}}}-m({{\bf{x}}})),$$

6 $${\Sigma }{*}=k({{{\bf{x}}}}{*},{{{\bf{x}}}}{*})-k({{{\bf{x}}}}{*},{{\bf{x}}})k{({{\bf{x}}},{{\bf{x}}})}^{-1}k({{\bf{x}}},{{{\bf{x}}}}{*}).$$

The mean of the predictive distribution, m*, provides the expected values of *f*(x*). The covariance of the predictive distribution, Σ*, provides the uncertainty estimates for the predictions. The GP models are implemented in Python using the gpflow package^39^.

Radial Basis Function (RBF) kernels are a widely recognised choice in GP models due to their ability to model a broad class of smooth functions that are infinitely differentiable, which often aligns well with many real-world phenomena. The RBF kernel is defined as:

7 $${k}_{{{\rm{RBF}}}}(x-{x}^{{\prime} })={\sigma }_{{{\rm{RBF}}}}^{2}\exp \left(-\frac{{\left(x-{x}^{{\prime} }\right)}^{2}}{2{\ell }_{{{\rm{RBF}}}}^{2}}\right).$$

The RBF kernel is stationary, meaning that the correlation between two points is a function of the difference in their inputs, $x-{x}^{{\prime} }$. It has two parameters, which are often referred to as hyperparameters within the context of the GP model. The lengthscale *ℓ*_RBF_ determines variability in the *x* direction, which affects the length of the wiggles in the function. The variance ${\sigma }_{{{\rm{RBF}}}}^{2}$ determines the average distance of the function from its mean.

While alternative kernels were considered, our aim here is not to identify the optimal kernel for this application. Instead, the focus is on demonstrating the value of GPs within a flexible, probabilistic forecasting framework for spatially disaggregated EV adoption. The RBF kernel was selected as a sensible default due to its versatility and suitability for modelling the smooth, cumulative growth patterns typically observed in adoption trajectories.

Since *ℓ*_RBF_ and ${\sigma }_{{{\rm{RBF}}}}^{2}$ are strictly positive, Gamma distributions, *Γ*(*α*, *λ*), are used as priors, where *α* is the shape parameter and *λ* is the rate parameter. The chosen priors for the RBF kernel hyperparameters are:

8 $${\ell }_{{{\rm{RBF}}}} \sim \Gamma \left(\alpha=10,\,\lambda=1\right),$$

9 $${\sigma }_{{{\rm{RBF}}}}^{2} \sim \Gamma \left(\alpha=3,\,\lambda=10\right),$$

where *Γ* is a gamma distribution. The lengthscale prior, *ℓ*_RBF_, has a mean of 10 years, which reflects a plausible range of temporal correlation for the model. Given that only 12 years of historical data are available, longer lengthscales are unlikely to be appropriate. The prior provides enough flexibility to capture varying temporal smoothness while discouraging too short-term or overly long-range dependencies.

The variance prior, ${\sigma }_{{{\rm{RBF}}}}^{2}$, has a mean of 0.3, allowing moderate variability in the GP function without being overly diffuse or concentrated around the mean. The likelihood noise variance prior, which represents observational noise present in the data, is set to ${\sigma }_{{{\rm{noise}}}}^{2} \sim \Gamma (\alpha=2,\,\lambda=100)$ with a mean of 0.02, allowing the GP to deviate moderately from the observed data, assuming the observations contain some noise. The noise variance is incorporated into the GP by adding ${\sigma }_{{{\rm{noise}}}}^{2}I$ to the kernel during inference, where *I* is the identity matrix.

To define the GP mean function, we fit a smoothing spline to the historical EVMS trajectory of each LAD. This provides a smooth, continuous representation of the broader adoption trend at the regional level, from which the GP then models neighbourhood-level deviations. The spline is fitted using the make_smoothing_spline() function from the scipy.interpolate module in Python^40^. This setup allows for flexibility: any scenario can be substituted into the mean function in place of the historical trajectory, enabling scenario-consistent forecasts while still capturing local variation through the GP kernel.

The probit transformation is defined as:

10 $$\,{{\rm{probit}}}\,(x)={\Phi }^{-1}(x)\,\,{{\rm{for}}}\,\,x\in [0,1],$$

where *Φ* is the cumulative density function (CDF) for the standard normal distribution. Due to the nature of the probit transform, an EVMS value of zero maps to negative infinity, which poses problems for model training. To address this, we remove all leading zeros from the training data. If no valid non-zero values remain, we substitute a single data point with a small positive value (0.0001), representing the most recent observation. Each GP model is then trained using all remaining non-zero EVMS data points.

We estimate a single set of GP hyperparameters shared across all neighbourhoods within a region. Let ${{{\mathcal{D}}}}_{i}=\{{{{\bf{x}}}}_{i},{{{\bf{y}}}}_{i}\}$ denote the training data for neighbourhood *i*, and let ***θ*** represent the shared hyperparameters (e.g., kernel lengthscale, variance, and likelihood noise variance). Suppose there are *N* neighbourhoods in total within the region. For each neighbourhood *i*, the GP model defines a marginal likelihood *p*(**y**_*i*_∣**x**_*i*_, ***θ***). We also place priors over the hyperparameters *p*(***θ***) to reflect prior knowledge and regularise the model. The posterior distribution over ***θ*** is proportional to the product of the likelihoods and the prior:

11 $$p({{\boldsymbol{\theta }}}| {{{\mathcal{D}}}}_{1},\ldots,{{{\mathcal{D}}}}_{N})\propto \left[{\prod }_{i=1}^{N}p({{{\bf{y}}}}_{i}| {{{\bf{x}}}}_{i},{{\boldsymbol{\theta }}})\right]p({{\boldsymbol{\theta }}})$$

Instead of computing this posterior directly, we perform Maximum A Posteriori (MAP) estimation by minimising the sum of the negative log posteriors (training losses) across all neighbourhoods:

12 $${{\mathcal{L}}}({{\boldsymbol{\theta }}})={\sum }_{i=1}^{N}\left[-\log p({{{\bf{y}}}}_{i}| {{{\bf{x}}}}_{i},{{\boldsymbol{\theta }}})\right]-\log p({{\boldsymbol{\theta }}})$$

This optimisation is performed using the L-BFGS-B algorithm^41^, as implemented in the scipy library^40^. Prior distributions are specified for each kernel hyperparameter to guide the optimiser toward reasonable values and improve convergence.

### Generating and interpreting forecasts

Since the GP is trained in probit space, predictions must be transformed back to the original EVMS domain for meaningful interpretation. While specific percentiles can be easily transformed, recovering the full predictive distribution is more complex. To address this, we draw 1000 sample trajectories from the GP, balancing computational efficiency with sufficient exploration of the distribution. These samples include observation noise to better reflect the uncertainty present in real-world data.

While the proposed model is not intended to identify causal drivers of EV adoption, the contribution of each model component to forecast behaviour and accuracy can be broadly characterised. The GP is autoregressive in time and combines three key elements: observed LSOA-level EV registration data, a regional EV adoption scenario used as the mean function, and a kernel with learned hyperparameters.

At short forecast horizons, predictions are dominated by the most recent LSOA-level observations, allowing the model to capture persistent local deviations from the regional trend. As the forecast horizon increases beyond the kernel lengthscale, the influence of historical local data diminishes, and the mean prediction gradually converges to the regional scenario. Consequently, long-term forecast accuracy is primarily governed by the accuracy of the regional scenario and how well it generalises to neighbourhood-level EV adoption, while short-term accuracy is driven by local historical data.

The kernel hyperparameters determine the degree of deviation from the mean function and the rate of convergence. In combination, these components allow the model to balance local adaptability with long-term consistency, while maintaining interpretable behaviour across time scales.

### Baseline models

To evaluate the performance of the proposed GP forecasting model, we compare it against three baseline approaches: scaled scenario, logistic growth, and linear extrapolation. Each baseline is designed to offer a plausible, lightweight alternative to more complex forecasting methods, while serving as a benchmark for assessing predictive accuracy.

The scaled scenario baseline modifies the regional-level EVMS scenario by scaling it to match each neighbourhood’s most recent observed EVMS in the training data. This approach preserves the shape of the regional scenario while adjusting for local differences in initial adoption levels.

The logistic growth baseline models each neighbourhood’s EVMS trajectory using the standard logistic function:

13 $$\,{{\rm{LG}}}\,(t)=\frac{1}{1+{e}^{-\kappa (t-{t}_{0})}},$$

where *κ* is the growth rate and *t*_0_ is the midpoint year. To constrain the fit and ensure consistency with broader trends, *κ* is restricted to within ±0.05 of the regional-level growth rate, and *t*_0_ must lie within ±5 years of the regional-level midpoint. These constraints allow for neighbourhood-specific flexibility while maintaining alignment with the regional scenario. Initial parameter estimates are derived from a logistic fit to the regional-level EVMS trajectory. The neighbourhood-level fits are then optimised using the curve_fit() function from the scipy.optimize module in Python^40^.

The linear extrapolation baseline projects neighbourhood-level EVMS forward using a simple linear trend fitted to the most recent three years of historical data. This approach aims to capture short-term dynamics. We implement this using the LinearRegression class from the sklearn.linear_model module in Python^42^.

### Deterministic evaluation metrics

Let ${\widehat{y}}_{i}$ be the forecasted EVMS for neighbourhood *i*, and *y*_*i*_ the corresponding observed value. Errors are aggregated across all neighbourhoods within a region *R*. The regional-level Mean Absolute Error (MAE) and Mean Error (ME) are defined as:

14 $${{{\rm{MAE}}}}_{R}=\frac{1}{| R| }{\sum }_{i\in R}\left|{\widehat{y}}_{i}-{y}_{i}\right|$$

15 $${{{\rm{ME}}}}_{R}=\frac{1}{| R| }{\sum }_{i\in R}\left({\widehat{y}}_{i}-{y}_{i}\right)$$

To enable fair comparison between regions with different average EV market shares, we normalise both metrics by dividing them by the regional mean observed EVMS:

16 $${{{\rm{nMAE}}}}_{R}=\frac{{{{\rm{MAE}}}}_{R}}{\bar{y}_{R}}$$

17 $${{{\rm{nME}}}}_{R}=\frac{{{{\rm{ME}}}}_{R}}{\bar{y}_{R}}$$

where ${\bar{y}_{R}}$ is the observed regional-level EVMS. The normalised mean absolute error (nMAE) measures the average forecast error as a proportion of the typical EVMS level in the region. The normalised mean error (nME) helps detect systematic bias, where values above zero indicate over-prediction, while values below zero indicate under-prediction.

Confidence intervals for nMAE are calculated using a bootstrap approach. This bootstrap approach resamples the original set of errors with replacement to generate *n* = 1000 synthetic datasets of equal size. For each resample, nMAE is computed to produce an empirical distribution of *n* = 1000 bootstrap estimates. The mean of this distribution provides a robust estimate of the metric, while the 95% confidence interval is obtained from the corresponding 2.5th and 97.5th percentile bounds.

### Reporting summary

Further information on research design is available in the Nature Portfolio Reporting Summary linked to this article.

## Supplementary information

Reporting Summary

Transparent Peer Review file

## Source data

Source Data

## Data Availability

The data that support the findings of this study are available at 10.5281/zenodo.19152368. The data generated in this study are provided in the Source Data file. Source data are provided with this paper.

## Code availability

The code used for the analysis in this study is publicly available in the GitHub repository: https://github.com/isaacflower/EVAdoptionForecastingFramework. An archived snapshot corresponding to the published version is deposited on Zenodo in: 10.5281/zenodo.19161363. Analysis and figure generation were performed using Python.
