# Supplementary material for: A probabilistic forecasting framework for neighbourhood-level disaggregation of electric vehicle adoption scenarios
Source: Nat Commun. 2026 Jun 13;17:7510. doi: 10.1038/s41467-026-74155-0 (PMC13408907; doi:10.1038/s41467-026-74155-0)
Supplement: Supplementary file 2 — Transparent Peer Review file [file 41467_2026_74155_MOESM2_ESM.pdf]

Peer Review File

A probabilistic forecasting framework for neighbourhood-level disaggregation of electric vehicle adoption scenarios

Corresponding Author: Dr Isaac Flower

This file contains all reviewer reports in order by version, followed by all author rebuttals in order by version.

Version 0:

Reviewer comments:

Reviewer #1

(Remarks to the Author)
In their manuscript "A probabilistic forecasting framework for neighborhood-level disaggregation of electric vehicle adoption scenarios", the authors introduce a new forecasting framework based on Gaussian processes that breaks down regional-level EV adoption scenarios to the neighborhood-level. They successfully validate their framework against several baseline methods, achieving moderate improvements. One possible application of the method is to inform a targeted extension of electric grids to accommodate the increasing power demand caused by EV charging.

The manuscript is well written, the figures are easy to grasp, and the validation appears thorough. I encourage the authors to publish their source code, as this would greatly help potential users of the framework. I only have a few minor remarks regarding the presentation and thus recommend accepting the manuscript for publication pending minor revision.

Minor comments

- Figures, generally: In printing, the grid lines of the figures are hard to make out. It would make the interpretation of the figures much easier if ticks were added to the x and y axes.
- P7: "...Linear extrapolation The details of these...." -- typo: missing period
- P8: "with significant improvement (at the 0.05 level) over all baselines" -- I assume that this refers to $p=0.05$ as a significance threshold? It might help the uninitiated reader to make this explicit.
- P8: "In contrast, the simpler baselines cannot incorporate such structured prior information, leading to larger forecast errors over time." -- maybe I'm mistaken, but doesn't the Scaled Scenario incorporate the LAD-level scenarios essentially as priors?
- P11: "DSOs" -- you introduce this abbreviation once on page 3, but repeating it on page 11 would improve readability
- P13: "organisations such as OFGEM and NESO" -- please explain these abbreviations/briefly mention what kind of organisation they refer to
- P15: "RBF kernels" -- similarly to "DSO", it would improve readability to repeat here what RBF means as it is mentioned just once back on page 4.
- P15: "alternative kernels such as the Matérn family" -- isn't the RBF kernel also part of the Matérn family?
- P16: In Eq (10), the likelihood noise variance σ_n^2 is specified. Yet, unless I missed it, it has not been introduced before. In the two paragraphs below, adding the corresponding symbols to the mentioned parameters (length scale prior, variance prior, likelihood noise variance prior) would improve clarity.
- P16/17: Generally, the derivation of the Gaussian process usage could be improved in clarity.
- P16, unnumbered equation at the bottom, and P17, unnumbered equation at the top -- N is not explicitly defined (number of neighborhoods).
- P17, unnumbered equation at the top -- $\mathbf X_i$ undefined.
- P17: Eq (12) -- typo: superfluous parentheses in the denominator

(Remarks on code availability)

Reviewer #2

(Remarks to the Author)
This study proposes a Gaussian Process (GP)-based forecasting framework to predict neighborhood-level EV registration, which could inform charging infrastructure planning in areas with limited fine-grained data. Below are my specific comments:

1. What is the purpose of clustering in your method? Are different Gaussian Process kernels applied to the various clustering results? If so, it is recommended to integrate the clustering method into your framework in Fig. 1. Additionally, how do different clustering strategies (e.g., varying the number of clusters or using alternative clustering methods) affect the accuracy of your results?

2. The paper proposes a methodology to predict the disaggregated level of EV share. From a methodological innovation perspective, it is not clear from the literature review how much research gap this work addresses. Similar prediction problems outside the EV domain could likely achieve higher accuracy using other machine learning approaches. In terms of generalizability, the proposed GP method is limited by computational efficiency, making it challenging to apply for larger-scale predictions. Moreover, the method does not provide more granular insights, such as neighborhood-level penetration rates for different types of EVs (e.g., BEVs, PHEVs). How could the method be improved to differentiate between these EV types?

3. Please explain more about the significance of the proposed methodology within the EV domain. Does this method have transferability to other contexts? How can the results be used to derive policy implications or provide insights for charging infrastructure planning?

4. To better address the question you proposed in Introduction (P2), “How might different neighborhoods collectively contribute to a regional adoption trajectory?”, should more neighborhood-level data be incorporated? On one hand, this could potentially improve model performance; on the other hand, it could help answer the why question regarding the neighborhood heterogeneity, thereby providing deeper insights.

(Remarks on code availability)
It is recommended that the authors provide a README file with clear instructions for installing and running the code. This would enhance the reproducibility and usability of the work.

Reviewer #3

(Remarks to the Author)
This is an interesting study regarding neighborhood level disaggregation of EV adoption. However, EV adoption has been widely studied and it’s hard to find any new insights offered by this study regarding EV adoption.
Detailed Comments
1. It is hard to identify any new insights provided by this study regarding EV adoption. Perhaps the contributions need to be explained well.
2. The data sets are described after the results. I would suggest describing the data along with the methodology prior to the results.
3. The datasets in section 4.1 do not provide the sample size and the summary statistics, which are critical.
4. The methodology in section 4.3 should explain why gaussian process was used instead of other rigorous frameworks.
5. It is not clear what input features are used for the models and what is the contribution of each factor towards the model accuracy.
6. The results for figure 6 and 7 needs to better explained.

(Remarks on code availability)
It seems fine with respect to the study

Version 1:

Reviewer comments:

Reviewer #1

(Remarks to the Author)
In the revised version of their manuscript, "A probabilistic framework for neighbourhood-level disaggregation of electric vehicle adoption scenarios", the authors have addressed all points raised during the first round of review.

I have only one minor comment: on pages 15 and 17, you have introduced the variance σ^2_{n}. I believe the index n stands for "noise"; however, it may also be interpreted as a numeric index. To avoid confusion, I'd recommend either removing the index, explaining it, or at least typesetting it non-italic, i.e., σ_n.

When this minor point is taken care of, I recommend the article be published.

(Remarks on code availability)

Reviewer #2

(Remarks to the Author)
All my concerns have been resolved. I have no further comments.

(Remarks on code availability)
I have no further comments.

Reviewer #3

(Remarks to the Author)
The authors have tried to address my comments from the previous round of review. However, some revisions are not satisfactory.

1. Response to comment 3 is not satisfactory. The authors should provide further details on the sample size and summary statistics rather than pointing towards confidence intervals.

2. Response to comment 5 is not satisfactory. Even though the authors claim that the goal is not to find causal driver, it is important to clarify the contribution of each input variable/factor towards the adoption and model accuracy.

3. Results section need better explanations and justifications rather than briefly mentioning what each figure is showing.

(Remarks on code availability)
It seems fine from the perspective of the paper.

Version 2:

Reviewer comments:

Reviewer #1

(Remarks to the Author)
In the second revised version of their manuscript, "A probabilistic forecasting framework for neighbourhood-level disaggregation of electric vehicle adoption scenarios", the authors have addressed the minor point I had raised in the previous round of review.

I have no further comments and recommend the article be published.

(Remarks on code availability)

Reviewer #3

(Remarks to the Author)
The authors have addressed the comments from the previous rounds in a satisfactory manner.

(Remarks on code availability)

Open Access This Peer Review File is licensed under a Creative Commons Attribution 4.0 International License, which permits use, sharing, adaptation, distribution and reproduction in any medium or format, as long as you give appropriate credit to the original author(s) and the source, provide a link to the Creative Commons license, and indicate if changes were made.
In cases where reviewers are anonymous, credit should be given to 'Anonymous Referee' and the source.
The images or other third party material in this Peer Review File are included in the article's Creative Commons license, unless indicated otherwise in a credit line to the material. If material is not included in the article's Creative Commons license and your intended use is not permitted by statutory regulation or exceeds the permitted use, you will need to obtain permission directly from the copyright holder.
To view a copy of this license, visit https://creativecommons.org/licenses/by/4.0/

We would like to thank the reviewers for their comments. Our responses to each comment are written below in red.

Reviewer 1

In their manuscript "A probabilistic forecasting framework for neighborhood-level disaggregation of electric vehicle adoption scenarios", the authors introduce a new forecasting framework based on Gaussian processes that breaks down regional-level EV adoption scenarios to the neighborhood-level. They successfully validate their framework against several baseline methods, achieving moderate improvements. One possible application of the method is to inform a targeted extension of electric grids to accommodate the increasing power demand caused by EV charging.

The manuscript is well written, the figures are easy to grasp, and the validation appears thorough. I encourage the authors to publish their source code, as this would greatly help potential users of the framework. I only have a few minor remarks regarding the presentation and thus recommend accepting the manuscript for publication pending minor revision.

Minor comments

1. Figures, generally: In printing, the grid lines of the figures are hard to make out. It would make the interpretation of the figures much easier if ticks were added to the x and y axes.

   Response: The figures have been updated with x- and y-ticks.

2. P7: "...Linear extrapolation The details of these...." -- typo: missing period

   Response: The period has been added.

3. P8: "with significant improvement (at the 0.05 level) over all baselines" -- I assume that this refers to $p=0.05$ as a significance threshold? It might help the uninitiated reader to make this explicit.

   Response: This line has been changed to:

   > *"...with statistically significant improvements (p < 0.05) over all baselines..."*.

4. P8: "In contrast, the simpler baselines cannot incorporate such structured prior information, leading to larger forecast errors over time." -- maybe I'm mistaken, but doesn't the Scaled Scenario incorporate the LAD-level scenarios essentially as priors?

   Response: Thanks for this observation – You are correct that the Scaled Scenario baseline does incorporate the LAD-level scenarios as a form of prior information. However, unlike the GP model, it does not converge back to the original LAD-level scenario over time. As a result, systematic errors accumulate, leading to forecast errors that grow with the forecast horizon. In contrast, the GP model explicitly incorporates the LAD-level mean function which provides a form of regularisation that the other baselines don't have. For clarity, we have rephrased the sentence in the manuscript to read:

   > *"In contrast, the simpler baselines do not benefit from the regularising effect of the LAD-level mean function used by the GP model, leading to larger forecast errors over time. While the Scaled Scenario incorporates the LAD-level scenario as prior information, it assumes that the historical proportional difference between LSOA- and LAD-level EV adoption remains constant over the entire forecast horizon."*

5. P11: "DSOs" -- you introduce this abbreviation once on page 3, but repeating it on page 11 would improve readability

   Response: Abbreviation has been repeated on page 13 for clarity.

6. P13: "organisations such as OFGEM and NESO" -- please explain these abbreviations/briefly mention what kind of organisation they refer to

   Response: The following sentence has been edited accordingly:

   > *"At the national level, organisations such as the Office of Gas and Electricity Markets (Ofgem, the UK energy regulator) and the National Energy System Operator (NESO, responsible for electricity system operation and planning) could use these forecasts to better understand..."*

7. P15: "RBF kernels" -- similarly to "DSO", it would improve readability to repeat here what RBF means as it is mentioned just once back on page 4.

   Response: RBF definition has been added on page 16 for clarity.

8. P15: "alternative kernels such as the Matérn family" -- isn't the RBF kernel also part of the Matérn family?

   Response: Thanks for pointing this out. The RBF kernel is a special case of the Matérn family where the smoothness parameter approaches infinity. The intention was to contrast the smooth RBF kernel with other commonly used kernels such as Matérn kernels with finite smoothness parameters (e.g., 3/2 or 5/2) that allow for rougher sample paths and may better capture adoption trends. To improve clarity, we have simplified the text to read:

   "*While alternative kernels were considered…*",

   removing reference to the Matérn kernels.

9. P16: In Eq (10), the likelihood noise variance σ_n^2 is specified. Yet, unless I missed it, it has not been introduced before. In the two paragraphs below, adding the corresponding symbols to the mentioned parameters (length scale prior, variance prior, likelihood noise variance prior) would improve clarity.

   Response: Corresponding symbols for the kernel hyperparameters have been added for clarity. As the reviewer highlights, the σ_n^2 parameter doesn't appear in the kernel equations. This is because it isn't technically a kernel hyperparameter and instead describes the observation noise present in the data. Therefore, a better description for this hyperparameter has been added and it has been separated from the others for improved clarity.

10. P16/17: Generally, the derivation of the Gaussian process usage could be improved in clarity.

    Response: We appreciate the reviewer's feedback and have revised this section to improve clarity. Specifically, we have added a new introductory subsection on regression (Section 4.3.1) to better set up the GP derivation and provide additional context. We have also included a new paragraph at the start of Section 4.3.2. explaining why Gaussian processes are particularly well-suited to this problem, helping to justify their use. In addition, we have made several minor edits to the derivation and supporting explanation to improve readability and flow.

11. P16, unnumbered equation at the bottom, and P17, unnumbered equation at the top -- N is not explicitly defined (number of neighborhoods).

    Response: Added equation number and this sentence to define N:

    "Suppose there are \(N \) neighbourhoods in total within the region."

12. P17, unnumbered equation at the top -- $\mathbf X_i$ undefined.

    Response: Added equation number. This is a good spot and was a typo that has now been corrected to x_i.

13. P17: Eq (12) -- typo: superfluous parentheses in the denominator

    Response: Extra parentheses have been removed.

Reviewer 2

This study proposes a Gaussian Process (GP)-based forecasting framework to predict neighborhood-level EV registration, which could inform charging infrastructure planning in areas with limited fine-grained data. Below are my specific comments:

1. What is the purpose of clustering in your method? Are different Gaussian Process kernels applied to the various clustering results? If so, it is recommended to integrate the clustering method into your framework in Fig. 1. Additionally, how do different clustering strategies (e.g., varying the number of clusters or using alternative clustering methods) affect the accuracy of your results?

   Response: The clustering is used solely to ensure a representative and diverse selection of local authority districts (LADs) for testing our forecasting model. By selecting two LADs per cluster, along with high and low outliers, we ensure that the evaluation captures a broad range of regional adoption patterns. The same GP forecasting framework is then applied to each selected LAD, with hyperparameters trained separately for each case.

Since the clustering step is only used to guide case study selection, it is intentionally kept separate from the core forecasting framework. In practice, LADs could be selected manually without impacting the methodology itself. As such, we did not experiment with alternative clustering strategies or numbers of clusters, as this was not the focus of the study. However, the clustering process remains a useful contribution, as it provides a systematic and transparent way to select test cases for future applications.

2. The paper proposes a methodology to predict the disaggregated level of EV share. From a methodological innovation perspective, it is not clear from the literature review how much research gap this work addresses. Similar prediction problems outside the EV domain could likely achieve higher accuracy using other machine learning approaches. In terms of generalizability, the proposed GP method is limited by computational efficiency, making it challenging to apply for larger-scale predictions. Moreover, the method does not provide more granular insights, such as neighborhood-level penetration rates for different types of EVs (e.g., BEVs, PHEVs). How could the method be improved to differentiate between these EV types?

   Response: As highlighted in the literature review, current approaches to EV adoption forecasting are limited by:

   (i) a lack of robust validation,

   (ii) insufficient spatial granularity, and

   (iii) inadequate treatment of forecast uncertainty.

   Our study addresses these gaps by introducing the first probabilistic forecasting framework applied at the neighbourhood level, providing forecasts with quantified uncertainty.

   While alternative machine learning techniques may perform well in other prediction contexts, their suitability for this problem has not been systematically explored. GPs were selected because they naturally quantify uncertainty, adapt well to limited data, and allow prior knowledge (e.g., regional adoption scenarios) to be incorporated. These characteristics are important for EV adoption forecasting but not easily replicated by many alternative methods. This work provides a foundation for future research, including the integration and benchmarking of other granular modelling techniques with uncertainty quantification.

   Although this case study focused on combined EV registrations to maximise data availability and simplify presentation, the framework is fully capable of forecasting BEVs and PHEVs separately. This requires updating the input vehicle registration data and the mean function scenario. We have clarified this point in Section 4.1. to make this flexibility explicit.

3. Please explain more about the significance of the proposed methodology within the EV domain. Does this method have transferability to other contexts? How can the results be used to derive policy implications or provide insights for charging infrastructure planning?

   Response: Previous studies have either focused on national- or regional-level forecasts instead of neighbourhood-level forecasts, relied on deterministic approaches that ignore uncertainty, or lacked validation against historical data. Our method is the first to generate probabilistic forecasts at a neighbourhood (LSOA) level, using detailed adoption data. This is the first study that uses the LSOA-level EV registration data from the UK's Department for Transport. To emphasise this novelty, we have added the statement:

   "*To our knowledge, this is the first academic study to use LSOA-level EV registration data to analyse or forecast EV adoption in the UK.*" *-* (1. Introduction, page 3)

   The framework has strong transferability potential. By substituting EV adoption data for other low-carbon technologies', such as heat pumps or rooftop solar PV, the same methodology could be applied directly. To highlight this, we added the following to the discussion section:

   "*Finally, the framework could be used to forecast the adoption of other domestic low carbon technologies, such as heat pumps or rooftop photovoltaic panels, if similar hierarchical adoption data is available.*" *-* (3. Discussion, page 14)

   From a policy and planning perspective, the outputs of this framework provide insights into the plausible range of future EV adoption at a highly localised level. This is directly relevant for:

   - Charging infrastructure planning - helping to anticipate future charger utilisation.

- Distribution network planning (particularly the last mile) - identifying where localised load growth may occur.
- Equity assessments - comparing adoption patterns across neighbourhoods to identify potential disparities in access to public charging or barriers to EV uptake.

The probabilistic nature of the forecasts enables decision-makers to weigh risks explicitly.

4. To better address the question you proposed in Introduction (P2), "How might different neighborhoods collectively contribute to a regional adoption trajectory?", should more neighborhood-level data be incorporated? On one hand, this could potentially improve model performance; on the other hand, it could help answer the why question regarding the neighborhood heterogeneity, thereby providing deeper insights.

   Response: We agree that incorporating additional neighbourhood-level data has the potential to improve forecast accuracy and provide deeper insights into the drivers of EV adoption. We now highlight this explicitly in the discussion section as an avenue for future research:

   "*There is also scope to enhance forecasting accuracy by incorporating additional features such as socioeconomic variables, housing stock characteristics, or infrastructure availability. Moreover, this could potentially provide deeper insights into the key drivers of EV adoption.*" - (Discussion, page 14)

Remarks on code availability: It is recommended that the authors provide a README file with clear instructions for installing and running the code. This would enhance the reproducibility and usability of the work.

Response: We have included a README file with instructions on how to install and run the code.

Reviewer 3

1. It is hard to identify any new insights provided by this study regarding EV adoption. Perhaps the contributions need to be explained well.

   Response: The key insight of this paper is how local EV adoption deviates from regional trends. This is made possible by the proposed EV adoption forecasting framework. Our unique approach overcomes the following limitations in the state of the art of EV adoption forecasting:

   (i) a lack of robust validation,

   (ii) insufficient spatial granularity, and

   (iii) inadequate treatment of forecast uncertainty.

   Our study addresses these gaps by introducing the first probabilistic EV adoption forecasting framework applied at the neighbourhood level, providing forecasts with quantified uncertainty. The focus is therefore on the methodology and its validation, rather than on identifying where EV adoption has occurred or will occur, and why.

   We believe the contributions of the study are already outlined towards the end of the introduction, but to make this clearer, we have added the following:

   "*To our knowledge, this is the first academic study to use LSOA-level EV registration data to analyse or forecast EV adoption in the UK.*" - (1. Introduction, page 3)

2. The data sets are described after the results. I would suggest describing the data along with the methodology prior to the results.

   Response: This is the format required by Nature Communications, which places the Methods section at the end of the paper. We have added one sentence in Section 2.1.1 to direct readers to the Methods section:

   "*Details of the datasets used for applying the framework to LSOAs and LADs in England and Wales, along with the data preprocessing steps undertaken, are provided in Section 4.1.*"

3. The datasets in section 4.1 do not provide the sample size and the summary statistics, which are critical.

   Response: The datasets listed in Section 4.1 are raw vehicle registration datasets, included primarily for reproducibility. These datasets cover all LADs and LSOAs in England and Wales, including the areas outside of the study area in this paper. The sample sizes and summary

statistics are already implicitly accounted for through the confidence intervals generated in the baseline comparison analysis.

4. The methodology in section 4.3 should explain why gaussian process was used instead of other rigorous frameworks.

   Response: We appreciate this suggestion. Justification for the use of Gaussian Processes (GPs) is provided in the introduction. Briefly, GPs were selected because they provide a flexible, non-parametric approach that can work with limited observed information and easily incorporate expert knowledge/judgement. In addition, GPs naturally perform uncertainty quantification, which can be updated as more information becomes available.

   In contrast, many alternative frameworks, such as neural networks, random forests, gradient boosting methods, and autoregressive models (e.g., ARIMA), either lack robust uncertainty estimation, require larger training datasets, or impose stronger assumptions about the data-generating process.

   We have added some further justification of our usage of GPs in Section 4.3.:

   > "*While many regression methods aim to learn a single best-fit function, GPs define a probability distribution over possible latent functions that could explain the observed data. This makes them particularly well-suited for sparse or small datasets, where uncertainty quantification is critical. Expert and prior knowledge can be easily incorporated into GPs, making them highly adaptable to various forecasting scenarios. Here, we use GPs to model the evolution of EVMS over time at the neighbourhood level, where each neighbourhood provides a univariate time series of EVMS observations.*"

5. It is not clear what input features are used for the models and what is the contribution of each factor towards the model accuracy.

   Response: The framework uses the following three inputs, described in section 2.1.1:

   > i) Observed neighbourhood-level EV registration data,
   >
   > ii) Regional EV adoption scenario, and
   >
   > iii) Choice of GP kernel and its hyperparameter priors.

   The EV registration data is converted into EV market shares, which are then used as the sole training dataset for the GP model. The regional scenario is incorporated as the mean function of the GP. The GP kernel's hyperparameters are optimised during training to minimise the model loss function.

   Because the objective of this study is to forecast local EV adoption, rather than to identify causal drivers, the model does not include a feature attribution component. However, extending the framework to include additional socioeconomic or infrastructure-related features is a valuable direction for future work, which is mentioned towards the end of the discussion section:

   > "*There is also scope to enhance forecasting accuracy by incorporating additional features such as socioeconomic variables, housing stock characteristics, or infrastructure availability. Moreover, this could potentially provide deeper insights into the key drivers of EV adoption.*" - (3. Discussion, page 14)

6. The results for figure 6 and 7 needs to better explained.

   Response: We have carefully revised the relevant sections and figure captions to ensure improve and readability.

   - Figure 6 illustrates the model's deterministic forecasting performance from an accuracy perspective (6a) and a systematic bias perspective (6b). 6a helps to show how much the framework reduces normalised mean absolute error over the raw LAD-level scenario.
   - To improve clarity of what the section is about, Section 2.3.2. has been renamed from "*Comparison with LAD scenarios*" to "*Forecast accuracy*".
   - The start of Section 2.3.2. has been edited to say: "*Here, we provide a more detailed evaluation of the GP model's forecasting accuracy across multiple forecast horizons for each LAD. To assess how much the proposed framework improves forecast accuracy, we use the idealised LAD-level scenario as a performance benchmark.*"

- The start of Section 2.3.3. has been edited to say: "*Figure 6b visualises systematic errors (bias) in the GP model's forecasts for each LAD by tracking the normalised Mean Error (nME) across different forecast horizons and starting points.*"
- Figure 6's caption has been updated to: "\textbf{a} Normalised Mean Absolute Error (nMAE) and \textbf{b} Normalised Mean Error (nME) of LSOA-level forecasts for each Local Authority District (LAD), evaluated across multiple forecast starting points (\(t_n\)) and target years (\(t_f\)). Shades of blue represent different forecast starting points, where forecasts made in \(t_n = 2018\) are evaluated for \(t_f \in \{2019, 2020, 2021, 2022, 2023\}\). Grey lines show the nMAE of the idealised LAD-level scenario used as a benchmark; values below this baseline indicate improved deterministic accuracy from spatial disaggregation. Deviations of nME from zero indicate systematic model bias, where positive values reflect over-prediction and negative values reflect under-prediction of EV adoption."
- Figure 7 illustrates the model's probabilistic performance—how calibrated the predictions intervals are. Therefore, Section 2.3.4. has been renamed from "*Prediction interval calibration*" to "*Forecast uncertainty calibration*" to improve clarity of what the section is about.
- Minor edits have been made to the first paragraph of Section 2.3.4 to improve clarity.
- Figure 7's caption has been updated to: "*Prediction interval coverage probability (PICP) plots for ten representative LADs over forecast horizons of*

  \textbf{a} \(h_f = 1\) year,

  \textbf{b} \(h_f = 3\) years, and

  \textbf{c} \(h_f = 5\) years.

  Each trace represents a forecast made at a starting year (\(t_n\)) and evaluated at a future year (\(t_f\)). The dashed diagonal denotes perfect calibration, where observed coverage matches the nominal prediction interval (PI) level. Traces above the diagonal indicate conservative forecasts (intervals too wide), while traces below indicate overconfident forecasts (intervals too narrow). Overall, the GP model achieves good calibration across LADs and horizons, demonstrating reliable uncertainty quantification."

Remarks on code availability: It seems fine with respect to the study

The code will be made available on Github with a README file.

We would like to thank the reviewers for their comments. Our responses to each comment are written below in red.

Reviewer 1

In the revised version of their manuscript, "A probabilistic framework for neighbourhood-level disaggregation of electric vehicle adoption scenarios", the authors have addressed all points raised during the first round of review.

I have only one minor comment: on pages 15 and 17, you have introduced the variance σ^2_{n}. I believe the index n stands for "noise"; however, it may also be interpreted as a numeric index. To avoid confusion, I'd recommend either removing the index, explaining it, or at least typesetting it non-italic, i.e., σ_n.

When this minor point is taken care of, I recommend the article be published.

Response: We thank the reviewer for highlighting this. We have changed the notation for the noise variance to σ_noise which should help to make it less ambiguous for the reader. This now appears on p16 and p18.

Reviewer 2

All my concerns have been resolved. I have no further comments.

Reviewer #2 (Remarks on code availability):

I have no further comments.

Reviewer 3

The authors have tried to address my comments from the previous round of review. However, some revisions are not satisfactory.

1. Response to comment 3 is not satisfactory. The authors should provide further details on the sample size and summary statistics rather than pointing towards confidence intervals.

Response: We have added a table (p8) containing key summary statistics for the datasets used in the analysis. For each of the 10 representative LADs used in the paper, the table shows the number of LSOAs (the sample size), and the median and interquartile range (IQR) of LSOA-level total vehicles, EVs, and EV market share (EVMS). This provides a sense of how many neighbourhoods (LSOAs) there are in each LAD, as well as the range of vehicle and EV adoption. We hope the addition of this table provides scientific value to the paper.

2. Response to comment 5 is not satisfactory. Even though the authors claim that the goal is not to find causal driver, it is important to clarify the contribution of each input variable/factor towards the adoption and model accuracy.

Response: We thank the reviewer for this clarification. We agree that it is important to explain what influences forecast behaviour and predictive performance. However, we would like to clarify that, in the proposed framework, the notion of a marginal “contribution” of individual input variables is not defined in the same way as causal models.

Our GP model is directly conditioned on historical LSOA-level EV adoption trajectories and does not contain explicit feature weights. Forecasting behaviour therefore emerges from the interaction between three model components rather than from separable input variables: (i) the observed LSOA-level data, (ii) the LAD-level scenario used as the GP mean function, and (iii) the kernel hyperparameters. As a result, predictive performance is largely driven by data availability, adoption stage, and learned correlation structure, rather than by identifiable causal factors.

To address the reviewer’s comment, we have expanded the qualitative interpretation of these components and their influence on forecasts. Specifically, we have added several new paragraphs to Section 4.3.8 (Generating and interpreting forecasts, p19) explaining how observed local data, the

regional scenario, and kernel hyperparameters respectively affect short- and long-horizon forecast accuracy. In addition, we now explicitly synthesise these effects at the end of the Results section (p14), stating that forecast performance is impacted by both historical data availability and the stage of local EV adoption. We believe this provides an appropriate explanation of what drives model predictions and accuracy.

3. Results section need better explanations and justifications rather than briefly mentioning what each figure is showing.

Response: We thank the reviewer for this comment. We have substantially revised the Results section to provide clearer explanations and justifications for the observed behaviour in Figures 6 and 7, rather than only describing their contents.

Specifically:

- In subsection *2.3.2 Forecast accuracy*, we now explain why forecast accuracy improves with later training periods, linking this behaviour to the autoregressive structure of the GP and improved estimation of kernel hyperparameters. We also clarify why the LAD-level baseline improves over time and why early models underperform at longer horizons due to overfitting to early adoption trends.
- In subsection *2.3.3 Forecast bias*, we provide a more mechanistic explanation for the generally low bias observed across LADs, relating this to the anchoring of the GP mean function to LAD-level EV adoption. We further explain the origin of systematic under- and over-estimation in specific LADs (e.g. Blaenau Gwent and Crawley) based on early zero-adoption patterns and rapid growth phases.
- In subsection *2.3.4 Forecast uncertainty calibration*, we expand the discussion to clarify how the GP captures uncertainty through both cross-LSOA heterogeneity and temporal variability within individual LSOAs, and how these effects are governed by the learned kernel hyperparameters.

These additions provide a clearer interpretation of the results and explicitly connect the observed performance to the underlying model structure and data characteristics.

Reviewer #3 (Remarks on code availability):

It seems fine from the perspective of the paper.
